# Gold Nanorods as Visual Sensing Platform for Chiral Recognition with Naked Eyes

**DOI:** 10.1038/s41598-018-23674-y

**Published:** 2018-03-28

**Authors:** Yanwei Wang, Xiaojuan Zhou, Chunli Xu, Yan Jin, Baoxin Li

**Affiliations:** Key Laboratory of Analytical Chemistry for Life Science of Shaanxi Province, School of Chemistry & Chemical Engineering, Shaanxi Normal University, Xi’an, 710062 P. R. China

## Abstract

Chirality plays a key role in modern science and technology. Here, we report a simple and effective sensing platform for visual chiral recognition of enantiomers. In this sensing platform, gold nanorods (AuNRs) prepared through a common synthesis route are used as colorimetric probes for visual recognition of glutamine (Gln) enantiomers. D-Gln could rapidly induce the aggregation of AuNRs, thereby resulting in appreciable blue-to-gray color change of AuNRs solution; however, L-Gln could not induce color change of AuNRs. This distinct color change can be easily distinguished by the naked eyes; as a result, a visual method of chiral recognition was suggested. The method was applied to determine the enantiometric excess of D-Gln through the whole range of −100% ~ 100%. The chiral assay can be performed with a simple UV-vis spectrometer or the naked eyes. Notably, the AuNRs do not need any chiral labeling or modification, and the chiral recognition is based on the inherent chirality of AuNRs. This chiral assay method is simple, sensitive, cheap and easy to operate. This study is the first example using AuNRs for direct visual recognition of enantiomers, and will open new opportunity to construct more chiral recognition methods for some important compounds.

## Introduction

Chirality is an intriguing natural phenomenon, and is also one of the basic characteristics of life system^1–3^. Most biologically important species (such as nucleic acid, proteins, drugs) are chiral. Consequently, many important biological processes involve specific interaction between chiral species, which governs the diffusion and distribution of biomolecules on natural membranes^1,2^. In most cases, the enantiomers of chiral molecules show obvious differences in toxicity, biochemical activity, metabolic mechanism and transport pathway. In general, only one enantiomer has the desired drug activity, and another exhibit no therapeutic effect and even may induce serious side effects. As a result, single-enantiomer drug ingredients are preferred over racemates. In order to ensure the validity of high-throughput studies, it is often necessary to measure enantiomeric purity in the synthesis of chiral compounds in daily practice. Thus, the detection method of enantiomers of chiral molecules is highly significant in the pharmaceutical and biochemical fields^3–14^. At present, chiral high-performance liquid chromatography, chiral gas chromatography and chiral capillary electrophoresis are commonly applied to detect enantiomers of chiral molecules^7,15^. But the separation methods require the expensive chiral column and involve tedious operations. Circular dichroism and optical rotation are able of rapid enantio-discrimination without the separation processes; however, the sensitivity of the two methods is relatively low, and the tolerance to impurities is low^16^. Nuclear magnetic resonance spectroscopy^17^ and mass spectrometry^18^ are also often used to detect enantiomers of chiral molecules, but the two methods require the expensive specialized equipment and the training of professional skills. Thus, it is very promising to establish a simple, sensitive and high-throughput method for detection of enantiomers. In chiral analysis, the biggest challenge is the visual recognition of enantiomers^19,20^. The realization of visual recognition requires the conversion of an enantioselective molecular recognition event into a considerable color change. However, very few examples of chiral recognition by apparent color-change have been reported^2,21^.

In recent years, one new kind of colorimetric methods has been developed based on the their unique surface plasmon resonance property of gold nanoparticles (AuNPs) or sliver nanoparticles (AgNPs)^22,23^. In AuNPs/AgNPs-based colorimetric assay, target can induce the obvious color changes of AuNPs or AgNPs solution, which is easy to be detected even by naked eyes. The AuNPs/AgNPs-based colorimetric assays have been proposed to detect many targets, including protein, DNA, RNA and small molecules^24,25^. However, until now, a few AuNPs/AgNPs-based colorimetric methods have been used in chiral measurement^26–35^. In order to achieve chiral recognition, the appropriate chiral ligand (such as *N*-acetyl cysteine, *L*-tartaric acid, *D*/*L*-penicillium, β-cyclodextrin, and nucleotides) is used to modify metal nanoparticles for obtaining chiral nanoparticles. In most of the reported AuNPs/AgNPs-based chiral recognition systems, the chirality of metal nanoparticles originated from the chiral ligands.

As a kind of elongated gold nanoparticles, gold nanorods (AuNRs) possess two resonance plasmon bands: the transverse resonance plasmon absorption peak at ca. 520 nm and the longitudinal resonance plasmon absorption peak in the visible and near-IR region. The longitudinal resonance plasmon absorption peak can be readily tuned by changing the aspect ratio, the medium dielectric content, or assembly^36^. Compared to spherical-shaped AuNPs, AuNRs are more sensitive to the micro-environment (such as interparticle distances, substrate, adsorbate and solvent)^37^. AuNRs have been used as signal elements for colorimetric sensing due to their strong distance-dependent optical property and their high absorptivity^38,39^. Moreover, recent studies showed that AuNRs exhibited chirality^40,41^. Maybe, AuNRs can act as colorimetric probes for chiral recognition. However, the field of enantioselective recognition based on color change of AuNRs still remains unexplored.

In this work, we reported one successful demonstration of AuNRs as visual sensing platform for chiral recognition. AuNRs were synthesized through the conventional chemical reduction process. Glutamine (Gln), an essential and important amino acid^42^, was chosen as model chiral molecule to evaluate the colorimetric discrimination enantiomers of AuNRs. Without any pre-treatment and prior derivatization, *L*- and *D*-Gln, respectively, was directly added into AuNRs solution. *D*-Gln could rapidly induce the appreciable blue-to-gray color change of AuNRs solution, whereas *L*-Gln could not induce color change AuNRs solution. So, the color change of AuNRs solution was used to recognize the chirality of Gln with the naked eyes. In this method, AuNRs don’t need any modifying with chiral compounds, and the inherent chirality of AuNRs is utilized. This study will also help people to understand the chirality of nanostructures^43^.

## Results

### Colorimetric response of AuNRs to chiral enantiomers

**Figure 1:**
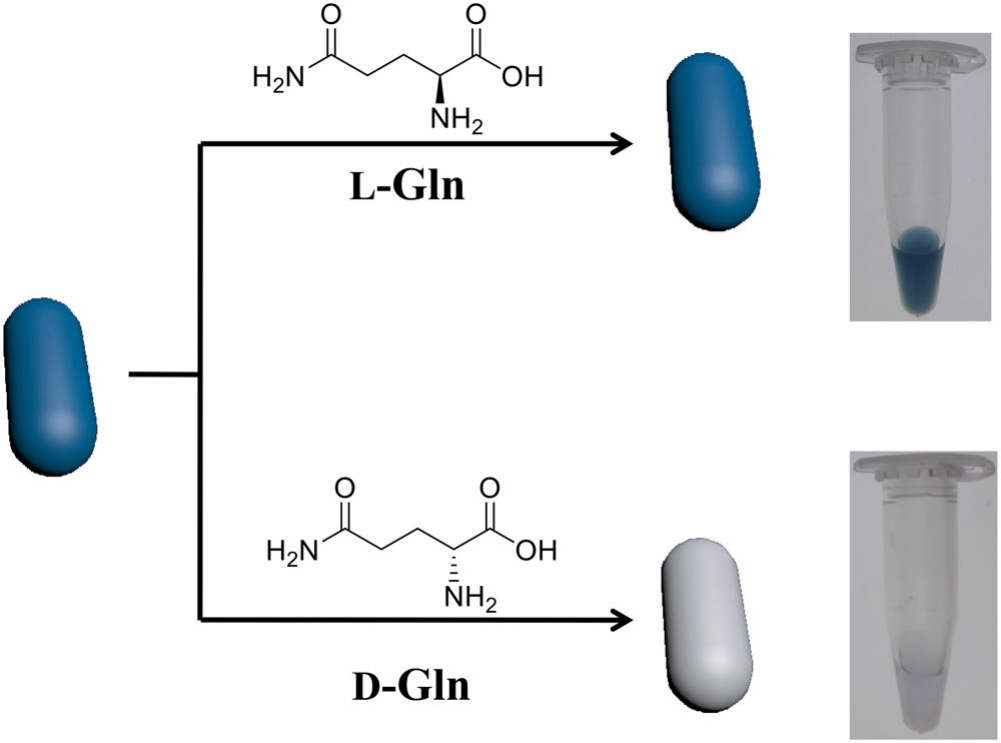
Schematic illustration of Gold nanorods as visual sensing platform for chiral recognition of L- and D-Gln.

**Figure 2:**
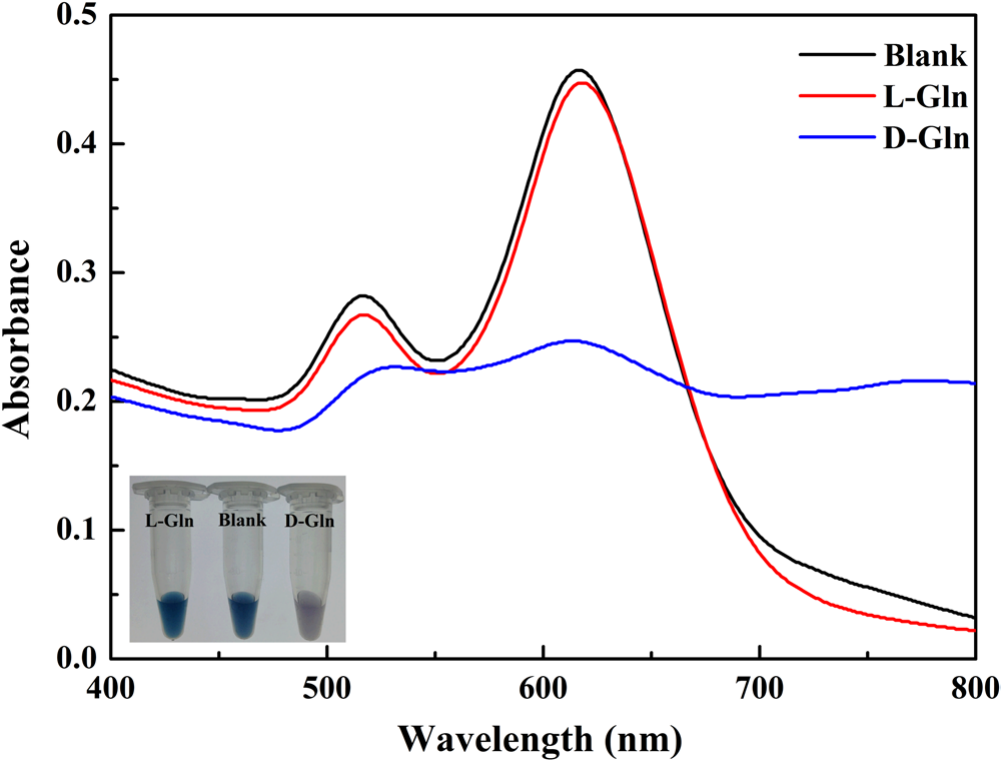
Absorption spectra of AuNRs in the presence of L-Gln or D-Gln. The inset shows the corresponding photographs. Experiment condition: 50 µL PBS buffer (pH 6.0), 50 µL AuNRs (0.24 nM), 100 µL L- or D-Gln (0.1 mM).

Figure 1 depicts the strategy for the colorimetric discrimination of chiral enantiomers using AuNRs as colorimetric probes. In this work, the conventional seed-mediated and CTAB-directed method was used to synthesize AuNRs. Due to the positively charged –NH_3_^+^ group of CTAB, the surface of CTAB-capped AuNRs has positive charges. Because of the electrostatic repulsion between positively-charged AuNRs, CATB-capped AuNRs solution is stable against aggregation. As shown in Fig. 2, the dispersed AuNRs have the longitudinal and the transverse plasmon resonance peaks at 620 nm and 517 nm, respectively. The two resonance peaks originate from the electron oscillation along the long and short axis, respectively^36,37^. Because of the strong resonance absorption of the dispersed AuNRs, the solution of dispersed AuNRs appear deep blue (as shown in Fig. 2). In this work, intriguingly, we found that the AuNRs displayed chiral-selective response upon addition of L-/D-Gln. The absorption spectra and color of the AuNRs solution are presented in Fig. 2. When adding D-Gln into the AuNRs solution, the absorption of AuNRs solution at 620 nm significantly reduced, and the solution color correspondingly changed from blue to gray. But, when addition of L-Gln, the absorbance spectra of AuNRs solution barely changed; at the same time, the color change of blue-to-gray wasn’t observed. Therefore, it is clear that the change in absorption spectra and solution color of AuNRs can be used to distinguish D-Gln from L-Gln.

**Figure 3:**
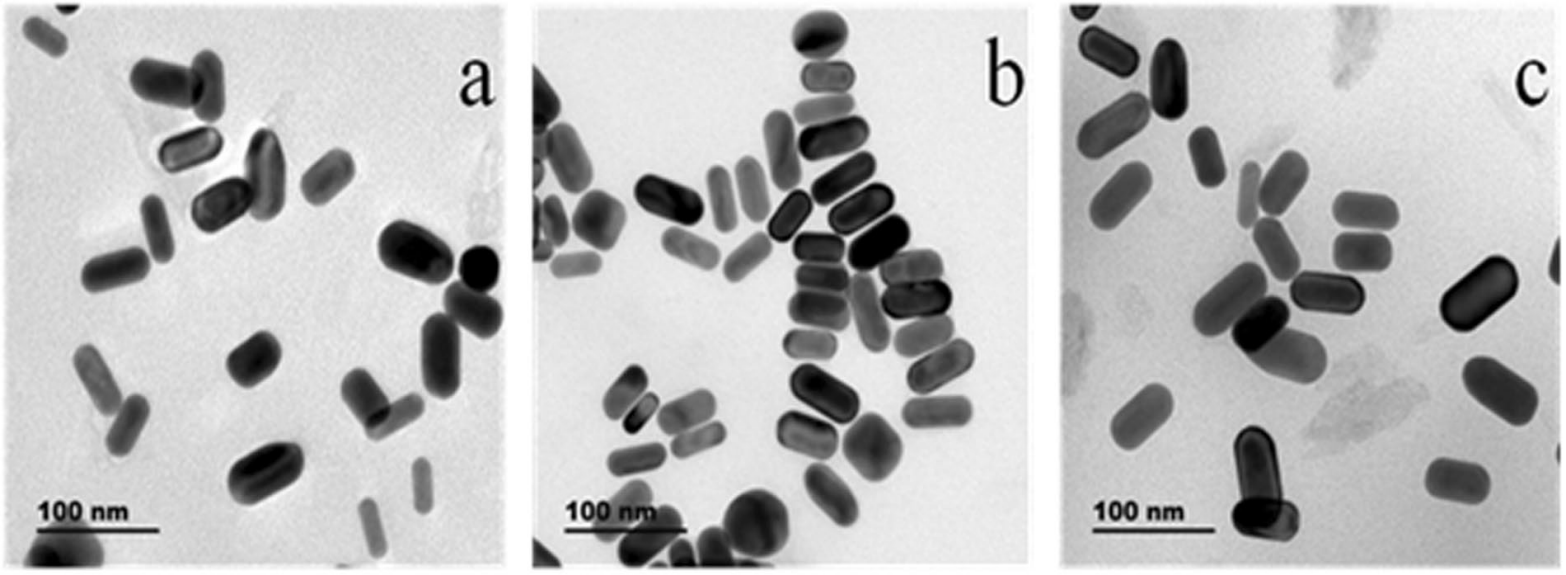
TEM images of AuNRs (**a**), AuNPs + D − Gln (**b**), and AuNPs + L − Gln (**c**).

The size and morphology of AuNRs before and after interaction with Gln were measured with transmission electron microscopy (TEM) images. The TEM images (Fig. 3) showed that the as-prepared particles were randomly dispersed, and the particles were mainly composed of the rod-shaped particles (Fig. 3a). These AuNRs had an average length of 52.31 ± 0.86 nm and an average diameter of 22.49 ± 0.56 nm, and the average aspect ratio was ca. 2.3. The AuNRs aggregated after interaction with D-Gln (Fig. 3b), while the AuNRs were monodisperse in the presence of L-Gln (Fig. 3c). For further verification of this phenomenon, dynamic light scattering (DLS) technique was used to explore the change of AuNRs induced by addition of D- or L-Gln. As shown in Figure S1, the hydrated size of AuNRs in the presence of 0.1 mM D-Gln was much larger than that of AuNRs, whereas the hydrated size of AuNRs was almost unchanged in the presence of 0.1 mM L-Gln. The DLS results were consistent with the TEM results (Fig. 3) and the absorption spectra (Fig. 2). The absorbance decrease at 620 nm was due to the aggregation of AuNRs^41^. Furthermore, the mixture solution of AuNRs and L- or D-Gln was centrifuged for 5 min at 1145 × g. As shown in Figure S2, some gray precipitate could be observed on the side wall of the tube, which contained D-Gln and AuNRs; no obvious change was found in the tube of L-Gln–AuNRs under the same experimental condition. The results indicated that D-Gln could result in the aggregation of AuNRs. In other words, D-Gln can be precipitated with AuNRs, whereas L-Gln cannot. The results reveal the potential of AuNRs to act as enantiospecific adsorbents for enantio-separation and enantio-purification.

### Optimization of experiment condition

To improve the chiral discrimination ability of AuNRs, some experimental conditions affecting response of AuNRs were optimized. We used different volumes of AuNRs stock solution to check the effect of AuNRs amount on the response of AuNRs in the presence of D-Gln or L-Gln. The results (Figure S3) showed that 50 μL of AuNRs stock solution (0.24 nM) could satisfactorily discriminate D-Gln against L-Gln. Media pH effect was examined in 4.0–8.0 pH range. The absorption change (Δ*A*_620_, Δ*A*_620_ = *A*_0_−*A*, *A*_0_ and *A* are the absorbance of AuNRs solution in the absence and presence of Gln, respectively.) versus media pH was obtained when fixing D-Gln and L-Gln concentration at 0.1 mM. When the pH of PBS buffer was 6.0, the sensitivity of the chiral recognition of Gln was the highest (Figure S4). The influence of incubation time from 1 min to 13 min was studied in detail. The results showed that D-Gln rapidly induced the aggregation of AuNRs, and Δ*A*_620_ of the system reached a maximum after 9 min incubation time. However, under the same experimental condition, L-Gln did not induce the aggregation of AuNRs. So, the incubation time of AuNRs and GLn was fixed at 9 min.

### Response of AuNRs to concentration of D-Gln or L-Gln

**Figure 4:**
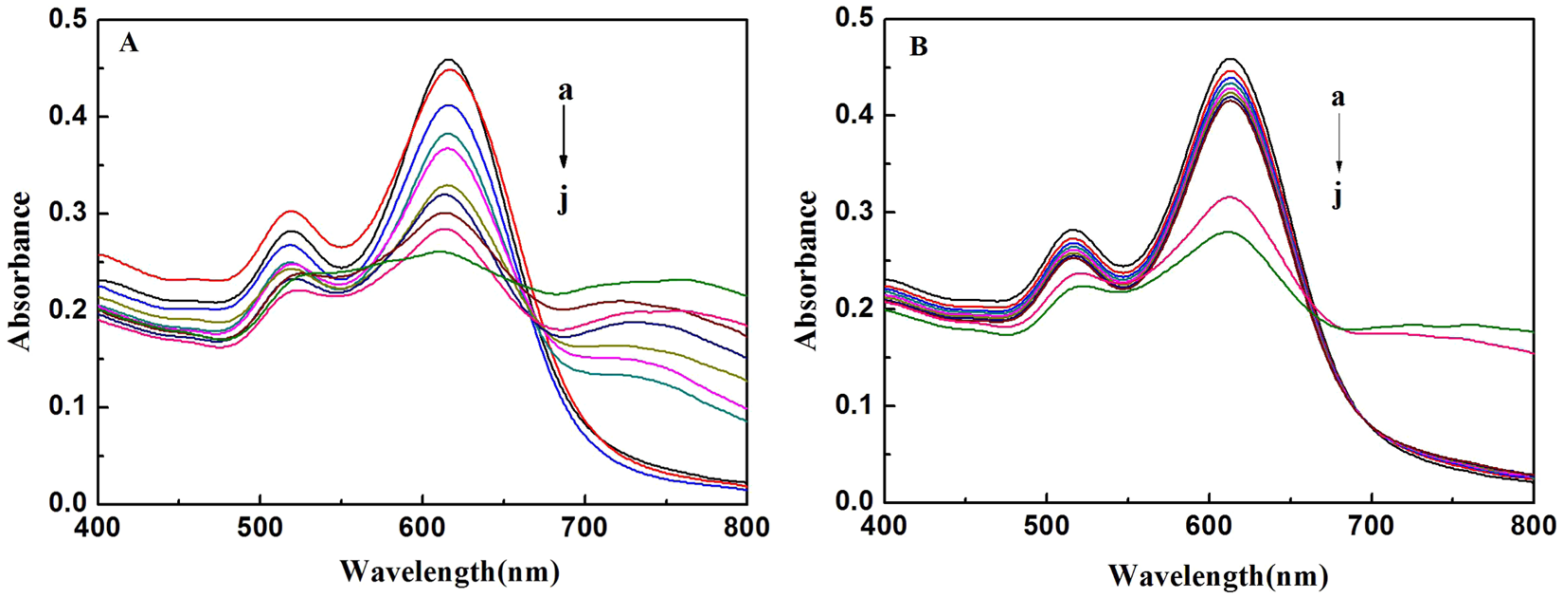
UV-vis absorbance spectra of AuNRs solution upon addition of D-Gln (**A**) or L-Gln (**B**) (a–j: 0, 0.003, 0.005, 0.01, 0.03, 0.05, 0.07, 0.1, 0.5, 1 mM). Experiment condition: 50 μL AuNRs, 50 μL PBS (pH 6.0), 100 μL D- or L- Gln.

To get a better understanding of the chiral recognition ability of AuNRs, the absorption changes of AuNRs caused by increasing concentration of Gln enantiomers were investigated. As shown in Fig. 4A, the absorbance at 620 nm gradually decreased with increasing D-Gln concentration from 0 to 1 mM. A sharp increase in the absorbance change (Δ*A*_620_) of AuNRs was observed with increasing D-Gln concentration from 0.003 mM to 0.1 mM (Figure S4). In distinguish contrast to AuNRs−D-Gln system, under the same experiment conditions, there was no distinct change of AuNRs spectra with addition of less than 0.5 mM L-Gln (Fig. 3B). The Δ*A*_620_ of AuNRs almost kept unchanged in the presence of less than 0.1 mM L-Gln; when L-Gln concentration was greater than 0.1 mM, the Δ*A*_620_ also increased with increasing L-Gln concentration (Figure S5). When the concentration was about 1 mM, the resulting Δ*A*_620_ of D-Gln was almost the same as that of L-Gln. The possible reason is that the high concentration of amino acid can make gold nanoparticles unstable. The resulting Δ*A*_620_ of 0.005 mM D-Gln was almost same as that of 0.1 mM L-Gln. This indicates that the Δ*A*_620_ of AuNRs induced by D-Gln is much greater than that by L-Gln. The obvious difference response of AuNRs to the two enantiomers suggests that the AuNRs can be used as colorimetric probe of detecting enantiomers of Gln.

### AuNRs-based colorimetric measurement of enantiomeric excess

**Figure 5:**
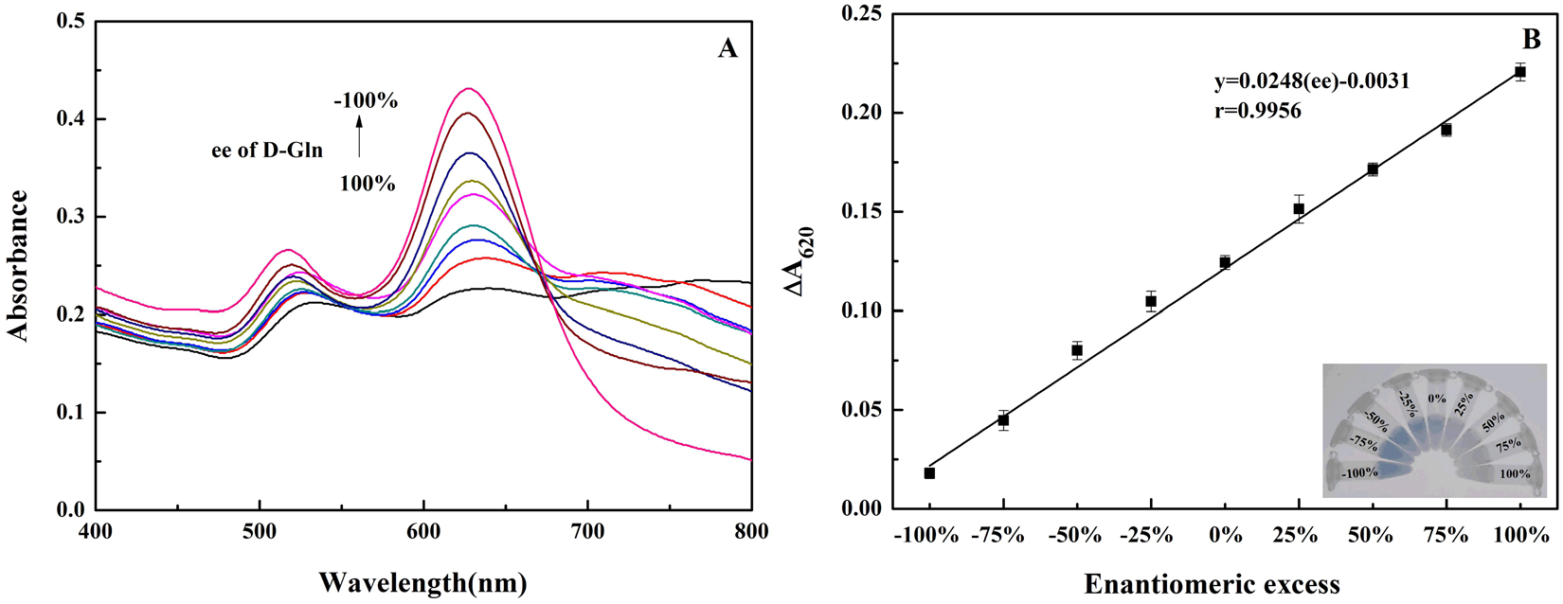
(**A**) UV–vis absorption spectra of AuNRs in the different enantiomeric excess of Gln (the total concentration of D- and L-Trp is 0.1 mM). (**B**) Plots of ΔA_620_ of the AuNRs versus the enantiomeric excess of Gln. Insert shows the corresponding photographs of AuNRs solution.

Enantiomeric excess (ee) is a measure for the purity of chiral substances, and ee is often used as one indicator of an asymmetric synthesis. The ee determination is of importance in some fields, such as production of chiral molecules and discovery of asymmetric catalyst^44^. AuNRs probes could discriminate Gln enantiomers. Thus, the AuNRs-based colorimetric assay may be used to determine Gln ee. In order to quantitative analysis of ee, all of experiments were performed under keeping the total concentration of the two enantiomers (D-Gln and L-Gln) the same. As shown in Fig. 4, the response of AuNRs to 0.1 mM D-Gln was rather big, whereas the response of AuNRs to 0.1 mM L-Gln was rather small. Therefore, the total concentration of Gln was chosen at 0.1 mM for ee determination. The Vis absorption spectra of the AuNRs solution in the presence of Gln with different ee values were measured. It can be seen from Fig. 5A that the absorbance at ca. 620 nm increased gradually with decreasing ee from 100% to −100%. The absorbance change at 620 nm (Δ*A*_620_) was linearly dependent on the ee of D-Gln in 100% to −100% range (Fig. 5B), and the correlation coefficient was 0.9956. Moreover, a color progressive change from blue to colorless was clearly observed with change of ee (Fig. 5B, inset). Furthermore, to testify the practical application of this chiral recognition, this AuNRs-based colorimetric method was used to determinate ee of D-Gln in some synthetic samples. For comparison, the chiral HPLC method (as the classic method for ee) was also applied to determinate the ee values in these same samples. The obtained results were shown in Table 1. The relative errors between two results were in the range from 5% to −5%, suggesting that the results from this AuNRs-based colorimetric method agreed with that from the chiral HPLC method. So, we have reason to believe that this AuNRs-based method can be used to detect ee with a simple colorimeter. It needs to mention that in this work the approach to ee determination was for D/L ratio, not for L/D ratio, due to the higher sensitivity of AuNRs toward D-Gln over L-Gln.

**Table 1:** Determination of enantiomeric excess of D-Gln in synthetic samples using the HPLC and this method.

Sample	ee with HPLC method (%)	ee with this method (%)	Relative error (%)
No. 1	−100.00	−99.97 ± 0.02	−0.03
No. 2	−74.76	−75.37 ± 0.04	0.82
No. 3	−51.26	−49.82 ± 0.08	−2.81
No. 4	−25.52	−26.05 ± 0.10	2.08
No. 5	−0.24	−0.25 ± 0.12	−4.00
No. 6	26.30	25.44 ± 0.15	−3.27
No. 7	50.88	50.08 ± 0.17	−1.57
No. 8	72.52	69.72 ± 0.19	−3.86
No. 9	100.00	99.50 ± 0.22	−0.50

### Colorimetric response of AuNRs to enantiomers of other α-amino acid

**Figure 6:**
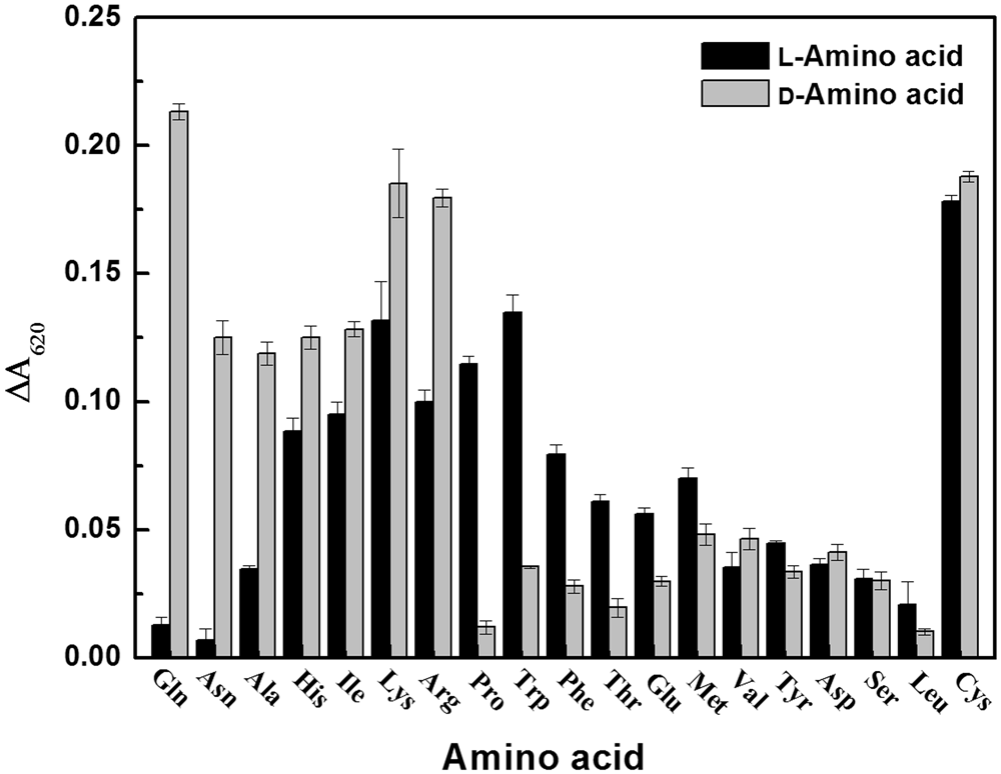
The absorbance change at 620 nm (Δ*A*_620_) of AuNRs in the presence of 1 mM *α*-amino acids. Experiment condition: 50 μL PBS (pH 6.0), 50 μL AuNRs (0.24 nM), and 100 μL amino acid.

To further investigated the chiral recognition ability of AuNRs, other 18 α-amino acids (asparagine (Asn), alanine (Ala), histidine (His), isoleucine (Ile), lysine (Lys), arginine (Arg), proline (Pro), tryptophan (Trp), phenylalanine (Phe), threonine (Thr), glutamic acid (Glu), methionine (Met), valine (Val), aspartic acid (Asp), tyrosine (Tyr), serine (Ser), cysteine (Cys) and leucine (Leu)) enantiomers were added into the AuNRs solution, respectively. As shown in Fig. 6, AuNRs exhibited a certain degree of difference to the enantiomers of *α*-amino acids except Cys. Under the above optimized condition, the Δ*A*_620_ was maximum between L-Gln and D-Gln, and so the AuNRs could effectively distinguish the enantiomer of Gln. Though the response differences of AuNRs were not remarkable for enantiomers of some *α*-amino acids (such as Asp and Tyr), chiral recognization performance of AuNRs for these *α*-amino acids may be improved through changing the experimental conditions (such as AuNRs’ size and media pH). For Cys, both of two enantiomers (L-Cys and D-Cys) could make AuNRs agglomeration. Among the 20 natural *α*-amino acids, Cys is only thiol-containing amino acid. Cys can bind on the surface of AuNRs *via* Au−S bond^45,46^, resulting in the aggregation of AuNRs. On the other hand, the clear changes in AuNRs solution color allow people to discriminate D- and L-amino acids through the naked eyes.

## Discussion

The mechanism clarification is one important step for recognition of chiral enantiomers. In most of these reported chiral recognition systems based on spherical nanoparticles^28−31,33−35,47^, chiral recognition depended on the presence of chiral ligands/stabilizer molecules. Recently, there are a number of studies on the chiroptical properties of AuNRs originating from the adsorbed chiral organic molecules or their assemblies induced by chiral templates^48,49^. For example, Liu group used the assemblies of AuNRs induced by Cys or glutathione for enantioselective circular dichroism sensing^48^. However, the chiral recognition based on the intrinsic chirality of the AuNRs is not be reported. In this system, the AuNRs are achieved *via* the seed-mediated and CTAB-directed method, and CTAB is capped on surface of AuNRs. As the capping agent of AuNRs, CTAB is achiral. In this work, the CTAB-capped AuNRs were acted as the chiral selector for discriminating Gln enantiomer. We infer that in this work the chiral recognition is the direct consequence of the intrinsical chirality of AuNRs. Metal surface exhibits the inherent chiral structure^50^, and Au nanoparticles or AuNRs can also exhibit intrinsic chirality^26,40,41,51–53^. In 2012, Bürgi group first gave the CD spectrum of Au clusters capped with the achiral ligand, which is the spectroscopic evidence for intrinsic chirality of Au cluster^54^. In this study, we measured the CD spectrum of CTAB-capped AuNRs, and CTAB-capped AuNRs exhibited a negative signal in CD spectrum (Figure S6). In order to further prove that AuNRs own an inherent chirality for visual recognition of Gln enantiomers, thioglycolic acid (TGA) was used to replace CTAB on the AuNRs’ surface. The results (Figure S6) showed that the CD spectra of AuNRs did not nearly change when TGA displaced CTAB on the AuNRs’ surface. This suggested that the chirality of CTAB-capped AuNRs did not come from CTAB (capping reagent). It is well known that the chiral enantiomer has specific rotation. So, we speculate that the CTAB-capped AuNRs should have optical rotation. The synthesis of the CTAB-capped AuNRs was repeated 10 times, and we measured the optical rotation of the obtained 10 solutions. As shown in Figure S7, the optical rotation of the 10 AuNRs solutions was always negative, and the average value was ca. (−) 2.8° for 0.24 nM AuNRs. That is to say, we got homochiral nanorods through the seed-mediated and CTAB-directed method without any other external chiral induction.

Chiral selectivity is mainly attributed to preferential interaction between one enantiomer and the chiral selector^42^. The optical rotation of D-Gln (5 mM) was measured to be (−) 4.647°, whereas that of L-Gln (5 mM) was measured to be (+) 5.433°. In this system, the CTAB-capped AuNRs exhibited a negative optical rotation, and the optical rotation of D-Gln is also negative. For the interaction between enantiomer and the chiral selector, the homochiral interaction is frequently stronger than the heterochiral interactions^28,34^. So, the binding action between D-Gln and CTAB-capped AuNRs is larger than that of the AuNRs and L-Gln due to the spatial requirements. As a consequence, CTAB-capped AuNRs exhibited good chiral selectivity to D-Gln.

The isoelectric point of Gln is 5.65, and Gln is negatively-charged in the pH 6.0 media. The CTAB-capped AuNRs are positively-charged. So, Gln can bind with CTAB-capped AuNRs *via* electrostatic interaction. To confirm the electrostatic interaction, the zeta potentials of AuNRs in the different conditions were detected. The zeta potential of 0.24 nM CTAB-capped AuNRs was +29.3 mV. After 0.5 mM D-Gln was added into this AuNRs solution, the zeta potential became about +0.07 mV. The zeta potential of CTAB-capped AuNRs was +22.5 mV in the presence of 0.5 mM L-Gln. These above experimental result demonstrated that the interaction between D-Gln and CTAB-capped AuNRs is much greater than that between L-Gln and CTAB-capped AuNRs. Furthermore, D-Gln decreased the zeta potential of the AuNRs surface form +29.3 mV to +0.07 mV, and then low surface charge density caused the aggregation of AuNRs. According to the above proposed mechanism for interaction between CTAB-capped AuNRs and D-Gln, we speculated that anionic AuNRs would not bind the negatively-charged D-Gln. To validate the scenario, the negatively charged AuNRs were prepared by depositing poly(sodium-4-styrenesulfonate) on CTAB-capped AuNRs^55^. The zeta potential of the anionic AuNRs was −40.8 mV. When D-Gln or L-Gln was added into the anionic AuNRs solution, the AuNRs did not aggregate (Figure S8). Thus, we reasoned that the decrease of the surface positive charge density of AuNRs resulted in the aggregation of CTAB-capped AuNRs.

## Conclusions

In summary, taking advantage of the intrinsic chirality of AuNRs, we presented here a sensing platform for chiral recognition. This sensing platform achieved the goal of translating one enanti-selective recognition process into an obvious color change. This chiral recognition is easily readout with a cheap UV-vis spectrometer and even the naked eyes. Of course, the test solution is colloidal, and the assay might also be useful for nephelometery. This study is the first example using AuNRs as colorimetric probes to construct visual sensing platform for chiral recognition with naked eyes, which gives new opportunity to design more efficient enantiosensing strategies. Furthermore, based on this study, AuNRs may be applied for biomolecule enantioselective transport.

## Methods

### Reagents and chemicals

Chloroauric acid (HAuCl_4_) was obtained from Shanghai Chemical Reagent Company (Shanghai, China). All of amino acids were obtained from Aladdin Chemistry Co. Ltd. (Shanghai, China). Silver nitrate (AgNO_3_), sodium borohydride (NaBH_4_), hydrochloric acid (HCl), cetyltrimethylammonium bromide (CTAB) and L-ascorbic acid were obtained from Sinopharm Chemical Reagent Company (Beijing, China). All other chemicals, unless mentioned otherwise, were of reagent grade. Milli-Q grade water was used throughout the experiment in this study.

### Preparation and characterization of AuNRs

In order to avoid some unwanted nucleation during the synthesis process and aggregation of metal colloid solution, all glassware and magnetic stirrer bars used in the following experiments were thoroughly cleaned in aqua regia (HNO_3_/HCl = 1:3, v/v), and then rinsed thoroughly in purified water. *Caution: great care has to be taken when handing aqua regia, which is a very corrosive oxidizing regent*. According to the reported protocol with necessary modification, AuNRs were synthesized *via* the seed-mediated and CTAB-directed method^56^. Firstly, the Au seed solution was prepared by mixing 5.0 mL 5.0 mM HAuCl_4_ and 5.0 mL 0.2 M CTAB aqueous solutions. Subsequently, 0.6 mL of freshly prepared and ice-cold NaBH_4_ solution (0.01 M) was added into the stirred mixture solution, and the color of the solution changed from yellow to brown. The above mixture solution was stirred for another 2 h, and then used or stored as the seed solution for synthetizing AuNRs.

The growth solution was prepared by mixing 5.0 mL 0.2 M CTAB solution, 5 mL HAuCl_4_ (1 mM) solution and 0.1 mL AgNO_3_ solution (4 mM) at room temperature. Then, during gentle stirring, 70 μL ascorbic acid (78.8 mM) was added to the above mixture solution. As a mild reducing agent, ascorbic acid can only reduce AuCl_4_^−^, and the growth solution changed from dark yellow to colorless. AuNRs with different longitudinal plasmon bands (different aspect ratio) can be acquired by adjusting the addition concentrations of AgNO_3_^56^. Finally, 12 μL of the Au seed solution was injected into the above growth solution, and the mixture solution was undisturbed for ca. 2 h at 30 °C. Excess CTAB was removed by centrifuging twice at 10000 rpm (15 min at one time). The supernatant solution was thrown away, and the resultant sediment was re-dispersed in pure water to obtain AuNRs solution. The AuNRs solution can be stable for at least two months under 4 °C. According to Orendorff and Murphy^57^, the concentration of as-prepared AuNRs was about 0.24 nM.

The UV-visible absorption spectrum of the prepared AuNRs solution was measured with one UV-Vis spectrophotometer (Hitachi U-3900H, Tokyo, Japan). CD spectrum was recorded on one Chirascan Applied Photophysics CD spectrophotometer (Leatherhead, UK). Optical rotation was measured with Autopol IV-T double wavelength automatic polarimeter (Rudolph, USA). TEM image was used to characterize the morphology of AuNRs by a JEM-2100 transmission electron microscope (Jeol Co. Ltd., Tokyo, Japan). The sample for TEM image was prepared through placing one drop of AuNRs solution on carbon-coated copper grid and then drying at room temperature (about 20 °C).

### Procedure of Colorimetric chiral recognition

Firstly, 50 μL of AuNRs (0.24 nM), 50 μL of pH 6.0 phosphate buffered saline (PBS, 0.01 M) and 100 μL of D-Gln or L-Gln with the appropriate concentrations were orderly added into one 1.5 mL eppendorf tube. Secondly, the above mixture solution was incubated at room temperature (about 20 °C) for 10 min. Finally, the UV-vis spectrum of the mixture solution was recoded; at the same time the color of colloid solution was recorded with a Cannon camera (500 digital).

## Electronic supplementary material

Supplementary Information
