# Supplementary material for: Gold Nanorods as Visual Sensing Platform for Chiral Recognition with Naked Eyes
Source: Sci Rep. 2018 Mar 28;8:5296. doi: 10.1038/s41598-018-23674-y (PMC5871867; doi:10.1038/s41598-018-23674-y)
Supplement: Supplementary file 1 — Supplementary Information [file 41598_2018_23674_MOESM1_ESM.doc]

Supporting information

**Gold nanorods as visual sensing platform for chiral recognition with naked eyes**

Yanwei Wang, Xiaojuan Zhou, Chunli Xu, Yan Jin and Baoxin Li*

Key Laboratory of Analytical Chemistry for Life Science of Shaanxi Province, School of Chemistry & Chemical Engineering, Shaanxi Normal University, Xi’an 710062, China. Email: libaoxin@snnu.edu.cn

**Figure S1.** Dynamic light scattering (DLS) curves of AuNRs (a), AuNRs+D-Gln (b), and AuNRs+L-Gln (c).

**Figure S2**. Photographs of the solutions of the AuNRs mixed with L- or D-Gln after centrifugation (4 000 rpm for 5 min).

**Figure S3.** Plots of absorption change of AuNRs upon the addition of D- or L-Gln (0.1 mM) at different volume of AuNRs (0.24 nM).

**Figure S4**. Effect of media pH on the chiral recognition of Gln. Experiment condition: 50 μL AuNRs (0.24 nM), 50 μL 0.01 M PBS, 100 μL D- or L-Gln (0.1 mM)

**Figure S5**. Plots of ΔA_620_ of AuNRs upon the addition of D-Gln or L-Gln at different concentrations.

**Figure S6.** CD spectra of CTAB-capped AuNRs solution and TGA-AuNRs solution (24 nM).

**Figure S7.** Optical rotation of 10 solutions of AuNRs


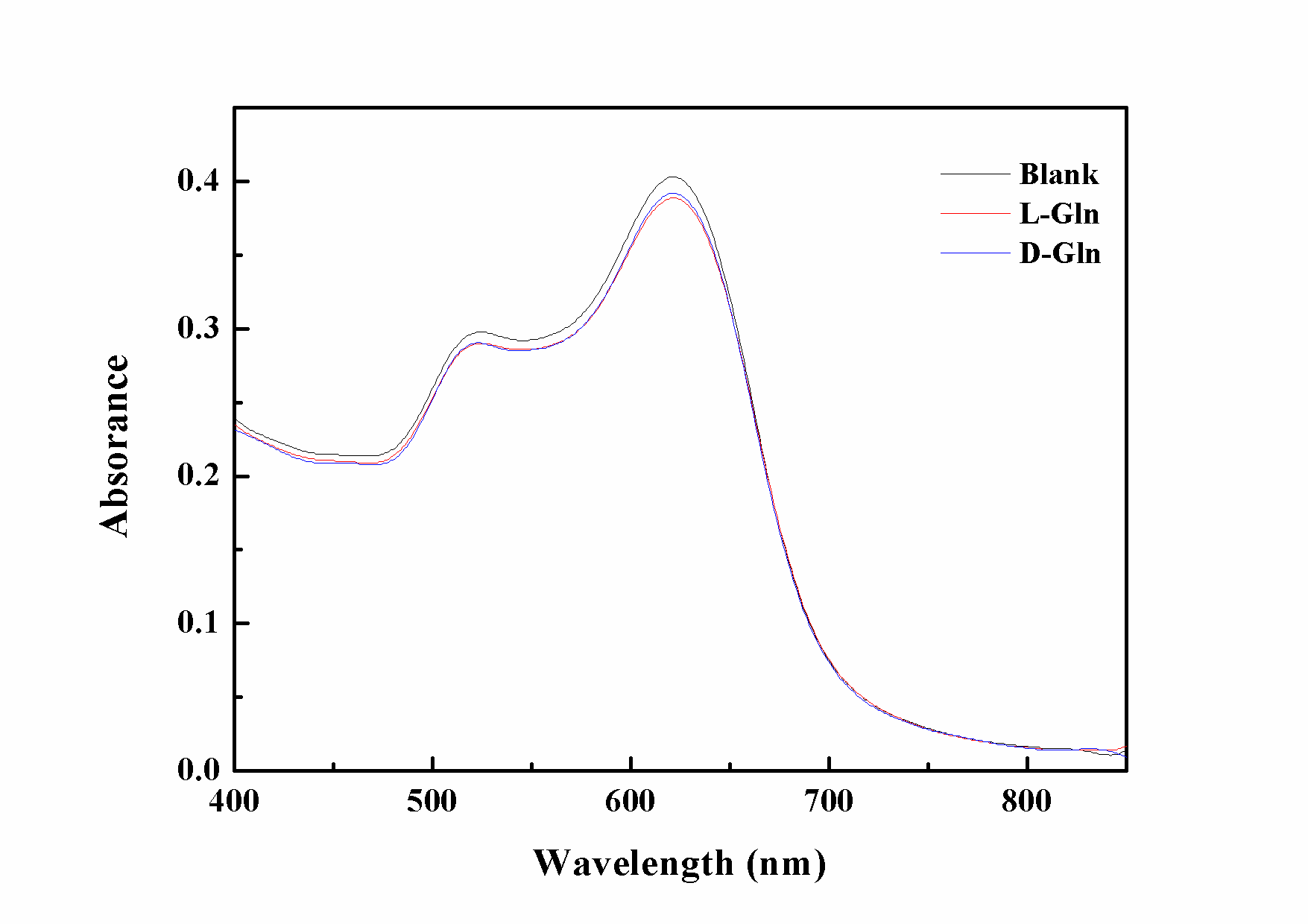


**Figure S8.** Absorption spectra of anionic AuNRs in the presence of L-Gln or D-Gln. The inset shows the corresponding photographs. Experiment condition: 50 µL PBS buffer (pH 6.0), 100 µL L- or D-Gln (0.1 mM).
